# Serological Survey of *Neospora caninum* and *Toxoplasma gondii* Co-Infection in Rodents in Northwestern Iran

**Published:** 2020

**Authors:** Naser NAZARI, Saeedeh SHOJAEE, Mahboobeh SALIMI, Mehdi MOHEBALI, Navid AHMADIFARD, Yazdan HAMZAVI, Zabihollah ZAREI, Reza FARAHMAND-RAD, Arezoo BOZORGOMID, Peyman HEYDARIAN

**Affiliations:** 1. Department of Medical Parasitology and Mycology, School of Medicine, Kermanshah University of Medical Sciences, Kermanshah, Iran; 2. Department of Medical Parasitology and Mycology, School of Public Health, Tehran University of Medical Sciences, Tehran, Iran; 3. Department of Microbiology, Asadabad School of Medical Sciences, Asadabad, Iran; 4. Department of Medical Parasitology and Mycology, School of Medicine, Qazvin University of Medical Sciences, Qazvin, Iran

**Keywords:** *Toxoplasma gondii*, *Neospora caninum*, Rodents, Indirect fluorescence antibody test (IFAT), Iran

## Abstract

**Background:**

Our knowledge of the epidemiology of rodents’ parasitic agents in Iran is scarce, although some of these pathogens play an important role in human and veterinary medicine, such as *Toxoplasma gondii* and *Neospora caninum*. The purpose of this study was to determine the seroprevalence of *Toxoplasma gondii* and *Neospora caninum* in rodents of northwestern Iran between Mar and Dec 2015.

**Methods:**

Overall, 157 serum samples from rodents (101 *Meriones persicus*, 41 *Mus musculus*, and 15 *Cricetulus migratorius*) were assayed by the indirect fluorescence antibody test (IFAT) for antibodies to *T. gondii* and *N. caninum*.

**Results:**

We found a prevalence of 20.38% (32/157) for *N. caninum,* 35% (55/157) for *T. gondii*. Co-presence of antibodies to *N. caninum* and *T. gondii* was found in 10 (6.36%) rodents. A significant association was found between the rodents species and seropositivity to *N. caninum* (*P*<0.05) but there was no association with rodents species for *T. gondii*. The overall prevalence of the aforementioned parasites was higher in male versus female rodents.

**Conclusion:**

The high seroprevalence of toxoplasmosis and neosporosis in rodents in the study area has implications for translocation of these infections across wider geographical regions since these rodents are mostly preyed on by cats or dogs; hence, which can transfer the parasite to other hosts.

## Introduction

Apicomplexan parasites are responsible for a wide variety of diseases in both humans and animals resulting in high economic losses such as the genera *Babesia*, *Theileria*, *Plasmodium*, *Eimeria*, *Toxoplasma*, *Neospora* and *Cryptosporidium* (1,2)*. Toxoplasma gondii* and *Neospora caninum* have similar life-cycles with different definitive hosts, the felids and canids, respectively, and have similar intermediate hosts including a wide range of mammals (3, 4). *Toxoplasma* is one of the most common zoonotic parasites and infected *one*-third of the total *world* population (5), while unlike toxoplasmosis, neosporosis is not recognized as an as zoonotic protozoan and only several studies have detected *N. caninum* antibodies in human (6, 7).

Rodents play an important role in the epidemiological chain of zoonotic pathogens such as *T. gondii* and *N. caninum* (8). Rodents are the largest group of mammals in the world, except Antractica, comprising more than 2,050 species on the earth (9, 10). Rodents can be a serious threat to the health of humans and animals. Although the role of rodents in the life cycle of neosporosis is unclear, rodents are exposed to infection because they cohabit with domestic animals and can ingest oocysts shed by dogs thus contribute to parasite distribution.

*N. caninum* have been reported in different hosts such as hooded crows (*Corvus cornix*) (11), stray dogs (12) and chickens (*Gallus domesticus*) (13) in Iran, but there is no reported survey on rodents. Furthermore, data on the prevalence of *T. gondii* infection in rodents in Iran are scant. However, seroprevalences of *T. gondii* and *N. caninum* infection in rodents is a good indicator for assessment of environmental contamination with oocysts shed by the definitive hosts (14).

Therefore, both *T. gondii* and *N. caninum* have a worldwide distribution and our knowledge of these species in Iran is limited, this study aimed to conduct a serosurvey of anti- *T. gondii* and anti- *N. caninum* antibodies in rodents living in Meshgin-Shahr County, northwest of Iran.

## Materials and Methods

### Study area

The samples were collected from Meshgin Shahr (38°23′56″N 47°40′55″E), a county in Ardabil Province, northwest of Iran. This province is considered as one of the coldest areas of Iran with a very cold weather for 5–8 months a year. Meshgin Shahr is a mountainous region at the hillside of Sabalan with several rivers.

### Animals and blood samples

Between, Mar and Dec 2015, 157 small rodents were sampled in Meshkin Shahr. Mice were trapped with live traps baited with fresh cucumber and walnut. The traps were set in the afternoon during trapping occasions and were collected in the next early morning. Morphological characteristics of every rodent and their sex were recorded. Blood samples were collected from rodents and were transported to the laboratory. After serum separation, it was labeled and stored at −20 °C until further use.

### Antigen preparation

The RH strain of *T. gondii* tachyzoites was prepared from the Dept. of Parasitology and Mycology, School of Public Health, Tehran University of Medical Sciences, Iran. Tachyzoites were propagated in the *peritoneal cavities of mice* and then harvested 3 d post-inoculation. They were then fixed in 1% formalin and washed three times with phosphate-buffered saline (PBS). Suspensions containing 50–100 tachyzoites per high power field (400×) were coated on glass slides. Antigen droplets were air-dried and stored at −20 °C. The NC-1 strain of *N. caninum* tachyzoites was prepared from Razi Institute, Shiraz, Iran. Antigen preparation of *Neospora* was the same as described above for *T. gondii* IgG-IFA

### Indirect fluorescent antibody test (IFAT)

For *Toxoplama*, the serum samples were diluted at 1:40, 1:50, 1:80, and 1:160 in phosphate-buffered saline (PBS). The exact cut-off values are determined by receiver operating characteristic (ROC) curve and Youden coefficient (15, 16). A titer of at least 1:40 was considered a positive result. For *Neospora*, the serum samples were diluted at 1:40, 1:50, and 1:80 in PBS and a titer of at least 1:50 was considered a positive result. The conjugate was made with 400 μL PBS, 10 μL Evans blue, and 1 μL goat anti-mouse IgG (Sigma-Aldrich, St Louis, USA). Anti-*T. gondii* antibodies were detected using the IFAT, as described previously (17). For each slide, a positive sample was used as a positive control and PBS was used as a negative control, and the results were determined in accordance with these controls.

This study was approved by the Ethics Committee of Kermanshah University of Medical Sciences.

### Statistical analysis

Data analysis was done with the SPSS software (ver.16, Chicago, IL, USA) using descriptive and inferential statistics. The Chi-squared and Fisher’s exact tests were used to report the associations between variables. *P*<0.05 was considered statistically significant.

## Results

One hundred and fifty seven small rodents, including 3 species of *Meriones persicus, Mus musculus* and *Cricetulus migratorius* were collected. The most abundant species in the present study was *Meriones persicus* (101/157). From 157 serum samples, 55 (35%) were positive for *T. gondii* (IFAT>40) and 32 (20.38%) were positive for *N. caninum* (IFAT≥1:50) (Table 1).

**Table 1:** Frequency of antibody titer against *T. gondii* and *N. caninum* by IFAT in rodents from Meshgin shahr County, Iran

***Titer***	***Toxoplasma gondii***		***Titer***	***Neospora caninum***					
	***Number of positive***	***Frequency (%)***		***Number of positive***	***Frequency (%)***
1:40	16	29.1	1:50	10	31.2
1:50	8	14.5	1:80	12	37.5
1:80	14	25.4	1:160	10	31.3
1:160	17	31	-	-	-
Total	55	100	Total	32	100

Seropositivity of toxoplasmosis in female rodents (25%: 15 of 60) was more frequent than that of males (17.5%: 17 of 97) (*P*=0.606). Moreover, 33% (32 of 97) of the males and 38.3% (23 of 60) of the females had anti-*N. caninum* antibodies. No significant difference between *N. caninum* infection and sex was found (*P*=0.309). The highest prevalence of Toxoplasma and Neospora infection was found in *Cricetulus migratorius* (5/15) and *Mus musculus* (16/41), respectively. A significant association was found between the rodents species and seropositivity to *N. caninum* (*P*<0.05). Table 2 presents the prevalence of infectivity with *T. gondii* and *N. caninum* in different rodents.

**Table 2:** Frequency of *Neospora caninum* and *Toxoplasma gondii* seropositive cases according to species of rodents

***Species***	***Number of rodents tested***	***Toxoplasma gondii***	**P *value***	***Neospora caninum***	**P *value***
		***Positive number [n (%)]***		***Positive number [n (%)]***	
*Meriones persicus*	101	33 (32.7)	0.596	13 (12.9)	0.002
*Cricetulus migratorius*	15	5 (33.3)		3 (20)	
*Mus musculus*	41	17 (41.5)		16 (39)	
Total	157	55 (35)		32 (20.4)	

The seroprevalence of Toxoplasma (32/55) and Neospora (17/32) infection was higher in male rodents versus female rodents. However, no significant association was found between gender and seroreactivity to *T. gondii* (*P*=0.606) or *N. caninum* (0.309). Co-presence of antibodies to *N. caninum* and *T. gondii* was found in 10 (6.36%) rodents.

## Discussion

Although parasite infections are widespread in nature and light infections are often asymptomatic, anthropogenic changes such as deforestation and urbanization can result in a loss of stability associated with altered pathogen growth, virulence and transmission rates. Therefore, baseline data on prevalence of parasitic infections in rodents are essential to provide an indicator of environmental health and to begin to assess and manage disease risks.

When the results of our study were compared with previous studies in different areas, the overall prevalence of *T. gondii* in the present study (35%) was higher than its prevalence in Iran (15%) (8), and Spain (12.4%) (18), but lower than its prevalence in England (40.78%) (19). No rodent had toxoplasmosis in Croatia (20). The overall prevalence of *N. caninum* was 20.38%, which was lower than its prevalence in Mexico (69.7%) (21). For Iran, there is no information on the prevalence of *N. caninum* in rodents. Vertical transmission may contribute to the maintenance of infection in this species (22). Another a probable reason for the high prevalence of infection could be the presence of infected definitive hosts. Previous studies of the seroprevalence of toxoplasmosis and neosporosis in dogs and cats in Meshgin Shahr County showed infection rates of 30.4% and 48%, respectively (23, 24). Furthermore, variations in the rate of seropositivity of *T. gondii* and *N. caninum* in different regions of the world can be attributed to the differences in diagnostic procedures.

Oocysts excreted in the environment by stray cats and dogs could be the direct or indirect source of contamination of rodents. Prevalence of *T. gondii* oocysts in environmental soil samples in Ahvaz (southwest of Iran) using PCR method is 9% (25). *Toxoplasma* DNA was found in 8.7% soil samples in Tehran, Iran (26).

The seroprevalence of *N. caninum* was higher in *Mus musculus* (41.5%) compared to *Cricetulus migratorius* (33.3%) and *Meriones persicus* (32.7%) (Table 1). Cohabitation of rodent with the definitive *hosts* of *N. caninum* is a putative risk factor for seropositivity due to high level of exposure to oocysts. In other words, rodent diet may not have an important role in the prevalence of neosporosis (27, 28).

In our study, gender seems to be a risk factor, with a higher seroprevalence of *T. gondii* and *N. caninum* infections in females than males. This agrees with the results reported in Ahvaz district, Southwestern Iran (29). Estradiol as female sex hormone induces suppression of natural killer cells (NK) cytotoxicity, one of the major sources of IFN-γ, and macrophages, responsible for IL-12 and IFN-γ production (30, 31). This explains the increased susceptibility of females to *T. gondii* and *N.caninum* infections.

## Conclusion

*N. caninum* infection occurs in all three species of rodents surveyed but at a much lower prevalence than *T. gondii*. To the authors’ knowledge, this is the first study of *N. caninum* in rodents from Iran. Further studies with larger populations should be carried out in urban, peri-urban, and rural areas of Iran to obtain a more detailed picture of the prevalence of both pathogens. In addition, future studies using molecular techniques are required to improve the detection rate of the parasites in rodents to promote our knowledge of the risks of zoonotic transmission.
